# Biological Implications and Functional Significance of Transglutaminase Type 2 in Nervous System Tumors

**DOI:** 10.3390/cells13080667

**Published:** 2024-04-11

**Authors:** Mariachiara Buccarelli, Giorgia Castellani, Vincenzo Fiorentino, Cristina Pizzimenti, Simone Beninati, Lucia Ricci-Vitiani, Maria Luisa Scattoni, Carlo Mischiati, Francesco Facchiano, Claudio Tabolacci

**Affiliations:** 1 Department of Oncology and Molecular Medicine, Istituto Superiore di Sanità, 00161 Rome, Italy; mariachiara.buccarelli@iss.it (M.B.); giorgia.castellani@iss.it (G.C.); lucia.riccivitiani@iss.it (L.R.-V.); francesco.facchiano@iss.it (F.F.); 2 Department of Human Pathology in Adult and Developmental Age “Gaetano Barresi”, University of Messina, 98125 Messina, Italy; vincenzo.fiorentino@unime.it; 3 Department of Biomedical, Dental, Morphological and Functional Imaging Sciences, University of Messina, 98125 Messina, Italy; cristinapizzimenti86@gmail.com; 4 Department of Biology, University of Rome “Tor Vergata”, 00133 Rome, Italy; beninati@bio.uniroma2.it; 5 Research Coordination and Support Service, Istituto Superiore di Sanità, Viale Regina Elena 299, 00161 Rome, Italy; marialuisa.scattoni@iss.it; 6 Department of Neuroscience and Rehabilitation, University of Ferrara, 44121 Ferrara, Italy; msc@unife.it

**Keywords:** transglutaminase 2, multifunctional enzyme, *TGM2* gene, crosslinking, nervous system tumors, glioma, neuroblastoma

## Abstract

Transglutaminase type 2 (TG2) is the most ubiquitously expressed member of the transglutaminase family. TG2 catalyzes the transamidation reaction leading to several protein post-translational modifications and it is also implicated in signal transduction thanks to its GTP binding/hydrolyzing activity. In the nervous system, TG2 regulates multiple physiological processes, such as development, neuronal cell death and differentiation, and synaptic plasticity. Given its different enzymatic activities, aberrant expression or activity of TG2 can contribute to tumorigenesis, including in peripheral and central nervous system tumors. Indeed, TG2 dysregulation has been reported in meningiomas, medulloblastomas, neuroblastomas, glioblastomas, and other adult-type diffuse gliomas. The aim of this review is to provide an overview of the biological and functional relevance of TG2 in the pathogenesis of nervous system tumors, highlighting its involvement in survival, tumor inflammation, differentiation, and in the resistance to standard therapies.

## 1. Introduction

Transglutaminase type 2 (TG2), also known as tissue transglutaminase (tTG), is a calcium-dependent acyltransferase, which catalyzes transamidation reactions leading to post-translational modifications of proteins, such as amine incorporation or formation of protein crosslinks. Since TG2 also shows a GTP binding/hydrolyzing activity acting as a G-protein, it is involved in signal transduction [1,2]. Importantly, the transamidation and GTP-binding activities are mutually exclusive [3]. Unlike other members of its family, TG2 is a ubiquitous enzyme, whose expression has been proved in various cell types in the central nervous system (CNS) [4] and is therefore considered to be responsible for the majority of transglutaminase activity in the brain [5,6,7]. TG2 regulates several physiological processes in the neural tissues, including nervous system development, neuronal cell death and differentiation, and synaptic plasticity [8,9]. Moreover, several studies have demonstrated that TG2 dysregulation may be involved in many pathological processes and degenerative disorders [5,10,11], including injury associated with ischemic stroke [12], Alzheimer’s disease [13], Parkinson’s disease [14], and Huntington’s disease [15]. Given its pleiotropic functions, it is not surprising that TG2 is involved in the initiation, survival, progression, tumor-related angiogenesis, and metastatic properties of several cancer types [16,17,18]; nervous system tumors are not an exception [19]. Interestingly, different studies reported that TG2 expression level is upregulated in CNS tumors compared to normal tissues [20,21,22,23,24,25]. However, TG2 expression in these tumors is heterogeneous [21,23,25]. Although brain and other CNS tumors are considered rare cancer types, their overall incidence has increased [26,27]. Malignant brain cancers constitute approximately 30% of the total but are responsible for the majority of the deaths; among them, glioblastoma (GBM) represents the most common and aggressive form of primary intracranial tumors with a median survival of approximately 12 months [28]. Therefore, a better understanding of the biochemical and molecular basis of brain tumors pathogenesis might open a new perspective in novel therapeutic approaches. Among peripheral nervous system tumors, neuroblastoma (NBL) is considered one the most common pediatric tumors, accounting for more than 15% of all cancer deaths in children [29]. Hence, the focus of this review is to describe the functional role of TG2 in the most studied nervous system tumors, taking into account its complexity and context-specific expression.

## 2. TG2 Description

### 2.1. TG2 Function

The transglutaminase (protein-glutamine gamma-glutamyltransferase; TG) family (EC 2.3.2.13) consists of a wide variety of highly conserved enzymes that are found ubiquitously expressed both in prokaryotic and eukaryotic organisms [30]. In humans, the TG family includes eight structurally and functionally related isoenzymes that catalyze the post-translational modification of proteins by the formation of isopeptide bonds [31]. Among them, the blood coagulation factor XIII (FXIII) is a zymogen whose active form is a TG [32]. TG1, TG3, and TG5 isoforms are primarily expressed in epithelial tissue, in which are responsible for cornified envelope assembly and for keratinocyte differentiation, contributing to the cutaneous barrier integrity [33,34]. TG4 is predominantly expressed in the prostate gland and is considered to be closely related to prostate cancer invasiveness [35]. TG6 is expressed in neurons [36], while to date the available information on the physiological role of TG7 is lacking. It is interesting to note that protein 4.2, the most abundant protein component of the erythrocyte membrane, shows high sequence homology with other TGs but is deficient in enzymatic activity [37].

**Figure 1:**
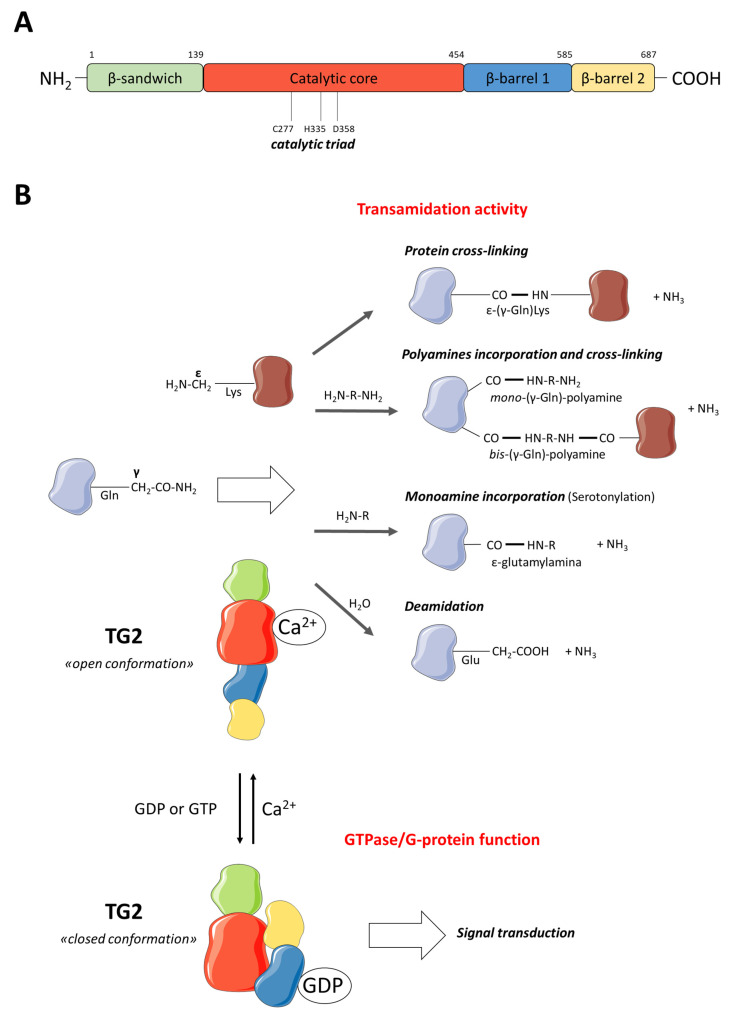
(**A**) Structure of TG2, composed of four domains: an N-terminal β-sandwich domain; an α/β-domain that comprises the catalytic triad; and two C-terminal β-barrel domains. (**B**) Reactions catalyzed by TG2: transmidation activity and GTPase/G-protein function (from Tatsukawa et al., 2016 [39] with modifications). Parts of the figure are drawn using pictures from Servier Medical Art (https://smart.servier.com (accessed on 13 January 2024)).

Compared to the abovementioned members of this family, TG2 is a multifunctional and ubiquitously expressed enzyme exerting transamidation (crosslinking, primary amine incorporation, and deamidation), protein disulfide isomerase (PDI), and serine/threonine kinase activities as well as G protein and cell surface adhesion functions [38,39,40]. TG2, a monomeric protein of 687 amino acids, consists of four domains: (i) an N-terminal β-sandwich that contains the binding site for fibronectin; (ii) an α/β-domain that comprises both the active site for transamidation activity with the catalytic triad (C277, H335, and D358) and the calcium-binding site; (iii) a C-terminal β-barrel domain that contains GTP/GDP-binding site; and (iv) another C-terminal β-barrel domain that can recruit and activate phospholipase C [18,41] (Figure 1A). As mentioned above, with the exception of protein 4.2, all TGs catalyze a calcium-dependent crosslinking reaction that starts with a nucleophilic attack, by the thiol of the active cysteine, of a γ-carboxamide group of a glutamine residue forming a thioester intermediate with ammonia release (Figure 1B).

Then, this intermediate reacts via the nucleophilic attack of an acyl acceptor (the ε-amino group of a lysine residue) leading to the formation of a covalent and degradation-resistant intra- or inter-molecular ε-(γ-glutamyl)lysine isopeptide bond [38,42]. A similar reaction also occurs with a large number of amine donors, including naturally occurring di- and polyamines such as putrescine (PUT), spermidine (SPD), and spermine (SPM), leading to the formation of either N-*mono*(γ-glutamyl)- or N,N-*bis*(γ-glutamyl)-PUT, -SPD, or -SPM [43]. If a water molecule is used as an acyl-acceptor, a deamidation reaction occurs, and glutamine residue is converted into glutamate residue [44]. The transamidation activity of TG2 depends on the presence of high calcium concentration. In fact, crystallographic data reveal that TG2 can assume at least two conformations with mutually exclusive functions. In the Ca^2+^-bound conformation, TG2 adopts an open (active) form with transamidation activity. When it binds GTP, TG2 shows a closed (inactive) conformation and acts as a G-protein contributing to transmembrane signaling [18,45] (Figure 1B). These two conformations determine different and, often, opposite effects on cancer fate depending on cell type and TG2 localization [46]. In particular, GTP-bound closed conformation exerts pro-survival functions, also in cancer stem cell models [47], while the open conformation may be considered, at least in some contexts, a tumor suppressor factor [48]. It has also been demonstrated that TG2 displays a PDI activity, which is principally involved in the formation of disulfide bonds in proteins of mitochondrial respiratory complexes [38,49]. Moreover, TG2 is involved in extracellular matrix (ECM) stabilization through a non-enzymatic activity. In fact, TG2 binds fibronectin in ECM acting as an integrin-binding coreceptor [50].

### 2.2. TG2 Gene Regulation

The human TG2 gene (*TGM2*; chromosome 20q11-12) expression is regulated by several factors according to numerous studies that have demonstrated the presence of regulatory elements in its promoter [3,51]. One of the first inducers of *TGM2* transcription to be studied was all-*trans* retinoic acid (RA), the major bioactive form of vitamin A, capable of binding the retinoic receptor (RAR) or retinoid X receptor (RXR), which in turn, after dimerization (RAR/RXR or RXR/RXR), binds response elements in *TGM2* promoter [52,53]. Moreover, TG2 expression and the activation of nuclear factor-kappa B (NF-κB) are linked by a molecular feedback loop [54]. Briefly, TG2 reduces the levels of IκBα (an inhibitor of NF-κB) through its crosslinking activity [55], whereas, in turn, NF-κB upregulates *TGM2* transcription (through its specific binding site in *TGM2* promoter) [56]. Interestingly, tumor necrosis factor (TNF)-α represents one of the most known inducers of NF-κB, thus it can be considered an inducer of *TGM2* transcription [57]. However, TG2 expression can be modulated by other mediators of inflammation like interleukin (IL)-1, IL-6, transforming growth factor (TGF)-β, and epidermal growth factor (EGF) [51]. The presence of hypoxic response elements (HRE) in the *TGM2* promoter is also described; thus, TG2 expression can be induced by hypoxia-inducible factor 1 (HIF1) [51,58]. It is therefore not surprising that, due to its complex regulation and all its enzymatic and non-enzymatic functions, TG2 is involved in several physiological and pathological processes [3,38,40,59].

It is interesting to highlight that GTP/GDP binding to TG2 depends also on alternative splicing of *TGM2* [60]. To date, five TG2 isoforms have been characterized. As described in the previous paragraph, the full protein (TG2-L; 687 amino acid residues) is encoded by a full-length transcript (TGM2_v1) as first reported by Gentile and coworkers [61]. The main trait of other not ubiquitously expressed alternative spliced forms is the loss of C-terminus to different extents, which impairs GTP binding, adhesion functions, and intracellular localization [60,62]. The TGM2_v2 spliced variant generated a short form of TG2 (TG2-S or TGH; 548 amino acids) first discovered in human erythroleukemia cells [63] and reported to be increased in brain tissue of Alzheimer’s disease patients [64]. Fraij and Gonzales also described a third variant (TGM2_v3) in RA-induced erythroleukemia cells, coding for a 349 amino acid protein (TGH2) [65], whose expression in macrophages was found to be associated with inflammatory state [66]. Two additional truncated isoforms, initially described in human umbilical vein endothelial cells (HUVEC), vascular smooth muscle cells and leukocytes, called tTGv1 (674 amino acid residues) and tTGv2 (645 amino acid residues) are encoded by TGM2_v4a and TGM2_v4b transcript variants, respectively [67]. Interestingly, it has been demonstrated that tTGv1 and tTGv2 are preferentially expressed in peripheral blood mononuclear cells derived from primary progressive multiple sclerosis patients [68].

The *TGM2* gene not only codes for several protein isoforms, but also includes two long non-coding RNAs (lncRNAs): (i) a lncRNA (TG2-lncRNA) within the first intron of the gene, whose expression correlates with TGM2_v1 [69] and (ii) a last exons variant (LEV) containing the last three exons and the 3′-UTR of the gene [70]. Although it has been suggested that lncRNAs have a role in the regulation of *TGM2* transcripts in some pathological conditions, this field is thus still open to future investigations [62,71,72,73].

## 3. Physiological Role of TG2 in Nervous System

TG2 plays several enzymatic and non-enzymatic functions in neurons, as mentioned above. Understanding how each TG2 function plays a role in neurons is a very hard task, which may be complicated further by taking into account whether TG2 works at intracellular or extracellular levels, within a specific subcellular compartment or moving across them [74]. Although TG2 was historically considered a cytosolic protein, the presence of fibronectin and integrin binding sites indicates important roles at the extracellular level [75], as well as its nuclear localization was strongly associated with neuron cell viability [76]. Nevertheless, a possible unifying function able to reconcile and explain most of these activities suggests that TG2 represents a stress response protein for several cell types, including neurons [40,77]. The peculiarity of neurons bears from their specific characteristic to represent the key cellular element to promote communication among cells, tissues, and organs. To achieve these results, neurons may communicate with other cells through synapses or neuromuscular junctions, where TG2 is deeply involved to induce stability of synaptic and neuromuscular junctions [9,78] and by crosslinking endogenous proteins [79]. Further, TG2 is involved in some hormone and cytokine secretion [40] as well as its enzymatic activity was also reported to inhibit neurotrasmitters release [80,81]. Moreover, the neurotransmitters may play a role not only as signaling molecules but also as additional post-translational modification, like protein serotonylation catalyzed by TG2 [82]. At the intracellular level, TG2 is involved in the covalent modification of several cytoskeleton elements [83] as well as acting as a G-protein [1,2,84] in many cellular models including NBL cells, with a bimodal effect [85] (see Figure 1B). In this regard, it is important to highlight that NBL cell lines are commonly used as neuronal models to investigate neurodegeneration and other neuronal disorders [86]. According to this evidence, although it is true that TG2 is considered a crucial player for CNS cells, its effects can be opposite in neurons or astrocytes survival after injury [87]. Similarly, it has been largely demonstrated that TG2 can modulate different physiological and pathological processes depending on the glial cell type [7]. Direct enzymatic action of TG2 on IL-2 was also demonstrated able to inhibit oligodendrocyte functions [88] as well as other studies demonstrated the TG2 involvement in oligodendrocyte differentiation [89] indicating a key role in neuroinflammatory mechanisms. The role of TG2 in induction of apoptosis, resulting in neuronal degeneration, is well established [90,91,92]. Therefore, its involvement in neurodegenerative diseases was reported [93,94]. Moreover, TG2 acts as an inhibitor of autophagy progression. The molecular mechanisms by which TG2 can modulate autophagy are still not well elucidated but it is likely that this enzyme may modify the cytoskeleton structure and function [95]. On the other hand, autophagic events in neuronal models have been demonstrated by showing crosslink of beclin 1 leading to autophagy regulation [96]. The role of a TG2-dependent midkine-homodimer formation in cerebellar neurons neurite outgrowth was also reported [97] as well as its role in neurite outgrowth mechanisms [98]. It is also important to highlight the involvement of 5-hydrioxytriptaminae (5-HT, serotonin) as an important neurotransmitter in many important functions like neuronal development and synaptogenesis as well as in several human diseases (see below). It is noteworthy that TG2 was demonstrated to specifically transamidates [^3^H]-serotonin to cell-surface proteins of glioma cells and the extracellular matrix-protein fibronectin [82]. The relevance of this reaction was previously reported also for the serotonylation of small G-proteins and their relevance in signal transduction pathways [99]. In this *scenario*, TG2 can be considered a key player in mediating neuron and glial cell functions and within the pathophysiology of CNS functions, by acting with several different molecular mechanisms [7].

All the above-reported cellular functions were studied and demonstrated by experimental approaches involving cellular or in vivo models, but stronger evidence about the direct involvement of TG2 within the above-described processes comes from the direct involvement of the enzyme or its enzymatic products. This is the case of the TG2 involvement in neurogenetic mechanisms demonstrated in a model of brain injury [100], in amyotrophic lateral sclerosis [101,102], in inflammation and multiple sclerosis [103,104] and in neurodegenerative diseases like Alzheimer’s [105,106,107,108], Parkinson’s [14,109,110,111], and Huntington’s disease [112].

In CNS and neurons the relevance of TG2 presence and activity indicates that it plays some key actions further demonstrated in pathological models where its involvement was extensively investigated and reported. Since one of the molecular characteristics of neurodegenerative diseases consists of the presence of insoluble protein aggregates, whose accumulation is often a TG2-dependent phenomenon, this is paving the way to the potential use of TG2 inducers and/or inhibitors as new therapeutic tools to treat such diseases [11,12,14,92,107].

## 4. Nervous System Tumors: Classification and Therapy

### 4.1. Peripheral Nervous System Tumors

Peripheral nervous system tumors are a morphological and biologically heterogeneous group of neoplasms that originate from different cell types. Reflecting the complexity of the tissue of origin, they can range from benign (e.g., schwannoma) to malignant (e.g., malignant peripheral nerve sheath tumors) forms [113]. To our knowledge, no sufficient data are available on the possible role of TG2 in the pathogenesis of these cancer types.

An exception is peripheral neuroblastic tumors (PNTs), a family of tumors arising from developing neural crest-derived cells of the sympathetic nervous system, which include peripheral NBL, ganglioneuroblastomas (intermixed and nodular), and ganglioneuroma [114]. NBLs (term often incorrectly used to define the entire PTN family) are embryonal tumors primarily developing in the adrenal gland (about 50% of cases) and in abdominal ganglia and represent the most commonly diagnosed cancer in pediatric age [115]. NBLs, as well as other PTNs, are characterized by the presence of different cellular components, including neuroblastic cells (at different grade of differentiation) and Schwann cells in different proportions. In particular, NBL are defined as neuroblastic Schwannian stroma-poor cancer [114,116]. This different cellular composition is also found in NBL cell lines, in which neuroblastic (N-type) and Schwann/mesenchymal (S-type) cells can be observed. Interestingly, it has been demonstrated that these two cell types may be derived from a distinct intermediate cell subpopulation (I-type) with stem-like properties and more tumorigenic potential [117,118,119]. The International Neuroblastoma Pathology Classification (INPC) has proposed a categorization of NBLs into three subtypes (undifferentiated, poorly differentiated, or differentiating) based on the differentiation state of neuroblastic cells [120,121]. An alternative and more recent classification, proposed by the International Neuroblastoma Risk Group (INRG) categorizes NBL into four risk groups [122]. Hence, the prognosis of patients with NBL is based on different parameters, including differentiation grade, genomic aberrations, ploidy, and *MYCN* (neuroblastoma MYC proto-oncogene/BHLH transcription factor oncogene) status [123,124]. In particular, *MYCN* represents the most studied gene in NBL and its amplification, which correlates with poor prognosis, can be found in approximately 25% of NBL patients [125,126]. Treatment strategies for NBL depend on the patient’s risk status and may include surgical resection, chemotherapy, radiation therapy, immunotherapy, and antiangiogenic therapy [127,128].

### 4.2. Central Nervous System Tumours

Tumors of the CNS represent a heterogeneous group of neoplasms with an incidence in the United States of 6.2 per 100,000 people per year and an estimated mortality rate of 4.4 deaths per 100,000 people per year from 2016 to 2020 [129]. In 2020, there were about 308,102 newly diagnosed cases of brain and other CNS tumors worldwide, resulting in an estimated 251,329 deaths [130].

**Figure 2:**
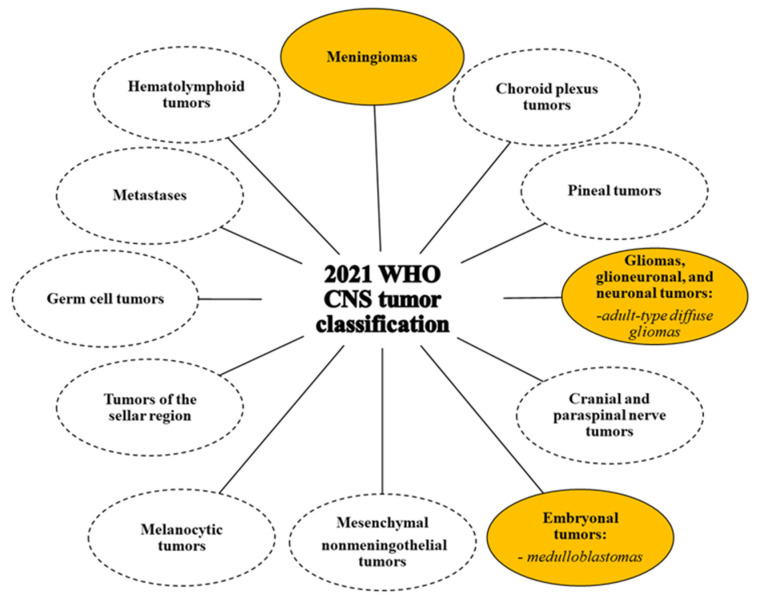
2021 WHO classification of the CNS tumors (fifth edition) [131]. Macrogroups of tumors according to the fifth edition of CNS tumor classification are reported. To date, the role of TG2 in CNS tumors has been studied in the macrogroups circled in yellow, as described in this review.

According to the most recent classification of CNS tumors developed by the World Health Organization (WHO) and published in 2021 (fifth edition), these tumors can be classified based on age of presentation, location, histological features, and molecular characteristics [131]. Particularly, histological features allow the identification of several macro groups including glial, glioneuronal, and neuronal tumors; choroid plexus tumors; embryonal tumors; pineal tumors; cranial and paraspinal nerve tumors; meningiomas; mesenchymal nonmeningothelial tumors; melanocytic tumors; tumors of the sellar region; germ cell tumors; hematolymphoid tumors; and metastases (Figure 2).

In particular, gliomas, glioneuronal, and neuronal tumors are the most common CNS tumors together with meningiomas and tumors of the sellar region. They originate from cells with glial differentiation capacity (neural stem or progenitor cells) and comprise astrocytomas, oligodendrogliomas, and ependymomas. All these neoplasms express glial markers, but they can be histologically distinct based on their architecture and type of growth. Whereas high-grade gliomas (such as GBMs) are characterized by a marked invasive phenotype, other low-grade gliomas (such as ependymomas or pilocytic astrocytomas) have a more circumscribed growth pattern. Among them, the most frequent is represented by GBM, the most malignant and aggressive form of brain tumor that accounts for the majority of brain cancer-related deaths [132]. GBM is a high-grade glioma and represents 14.3% of all CNS tumors and 49.1% of CNS malignant tumors [133].

Embryonal CNS tumors such as medulloblastoma (MB) and CNS neuroblastoma (CNS-NBL) are typical of pediatric age and include a wide range of locations and from low to high aggressive behaviors. However, according to the 2021 WHO CNS tumor classification, histological features are not sufficient to classify CNS tumors, in particular for glial and embryonal tumors, and the determination of specific molecular alteration is required [131,134]. In fact, over time, multiple molecular alterations have been identified with important prognostic and predictive implications. These molecular markers, partly associated with specific histological characteristics, can define diagnostic categories with significantly different prognoses, being more effective than morphological parameters alone. The main molecular alterations were widely described by Berger and colleagues [134].

Based on this evidence, the 2021 WHO CNS tumor classification provides a nosological classification that combines both phenotypic (i.e., morphological) and genotypic parameters to reach an integrated histological-molecular diagnosis [131]. Based on this classification, the expected grades of CNS tumors range from grade 1 (benign neoplasm, potentially curable with surgery alone) to grade 4 (highly malignant neoplasm). This updated version expands on the previous by emphasizing molecular markers in both classification and grading, and it incorporates the majority of the recommendations made in the cIMPACT-NOW publications [135]. The classification of adult type diffuse gliomas has been optimized to include only three types, including astrocytoma, *IDH* (isocitrate dehydrogenase) mutant (grades 2, 3, or 4), oligodendroglioma, *IDH* mutant and 1p/19q codeleted (grades 2 or 3), and GBM, *IDH* wild type (grade 4) [134]. Moreover, the MB definition makes use of both histological and molecular classification separately, without combining the corresponding criteria [136].

It is important to note that brain tumor classification is a dynamic field, and ongoing research continues to refine our understanding of the molecular and genetic characteristics of different tumor types. This knowledge is essential for developing personalized treatment strategies and improving patient outcomes. The inclusion of molecular markers has in fact improved diagnostic accuracy and allowed the development of targeted therapies based on genetic anomalies of specific tumor types [137]. Therapies for CNS tumors may include surgery, radiation therapy, chemotherapy, targeted therapies, and immunotherapy in a multidisciplinary approach [138,139].

For low-grade, well-circumscribed tumors, total resection in combination with radiotherapy, is associated with better overall survival (OS) and excellent progression-free survival (PFS) [140]. Maximal surgical resection, followed by radiotherapy plus concomitant and maintenance chemotherapy with temozolomide (TMZ), is more indicated for high-grade gliomas such as GBMs and MBs, although almost all patients experience tumor progression and worst OS [141,142].

Based on the classification of nervous system tumors previously reported, in the following paragraphs we described the role of TG2 in the context of pathogenesis of these tumors, in particular its involvement in radio- and chemoresistance mechanisms in GBMs and meningiomas, tumor inflammation in medulloblastomas, and differentiation in NBLs and GBMs.

## 5. TG2 in Adult-Type Diffuse Gliomas

It has been reported that upregulated expression of TG2 is associated with high-grade adult-type diffuse gliomas and correlates with poor patient survival [23,25,143]. These observations led to an increasing interest in investigating the role of TG2 in high-grade gliomas, particularly GBM. The first observation of TG2 increased expression in tissue sections of GBM was reported in the study of Iwaki and colleagues [20]. Via immunohistochemical analysis of fifty-eight brain tumor surgical specimens, they showed that the presence of positive immunoreactivity tends to increase in malignant counterparts. Particularly, TG2 expression was detected in GBM cells palisading along the periphery of the necrosis [20]. This result was confirmed by the study of Hilton and colleagues, which also reported a strong TG2 labeling of endothelial cells in GBM is as intense as that in pseudopalisading tumor cells, especially in vessels showing microvascular proliferation [21]. In GBM cell lines, an increased expression of TG2 was also reported, although its role in the proliferative process was dependent on the cell type [144,145]. The crucial role of TG2 in promoting gliomagenesis has been demonstrated by Zhang and colleagues [25]. They identified a novel functional interplay between TG2, EGFR, and brain tumor progression, dependent on TG2 GTP-binding capability. Particularly, they showed that TG2 enhanced EGFR activation and downstream signaling activities through its association with the E3 ubiquitin ligase c-Cbl, thus preventing EGFR ubiquitylation [25]. These findings lead to more and more interest in investigating TG2 as a potential marker of tumor aggressiveness, and consequently as a promising therapeutic target.

**Figure 3:**
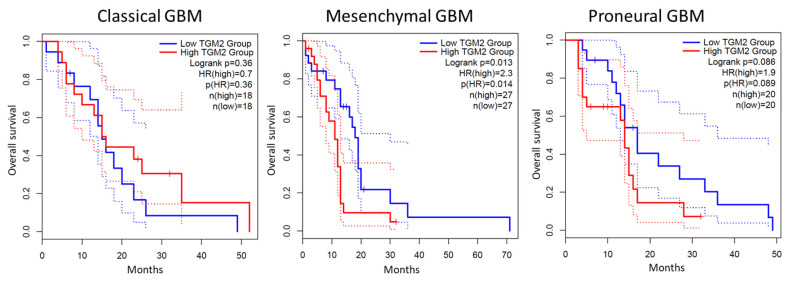
Kaplan–Meier survival curves in the three GBM subtypes: classical (**left panel**), mesenchymal (**center panel**) and proneural (**right panel**), using GEPIA2 database (http://gepia2.cancer-pku.cn/#index) (accessed on 15 January 2024) [153]. The median is selected as a threshold for separating high TG2 expression and low TG2 expression groups. No statistically significant differences were observed between high TG2 expression and low TG2 expression groups in the cohort of classical (*n* = 36, *p* (HR) = 0.36; (**left panel**)) and proneural (*n* = 40, *p* (HR) = 0.089; (**right panel**)) GBM patients. High expression of TG2 significantly correlates with a poor clinical outcome in the cohort of mesenchymal GBM patients (*n* = 54, *p* (HR) = 0.014; (**center panel**)).

Recently, it has been reported the finding of a correlation between TG2 expression and GBM subtypes [24,143,146,147]. Based on a transcriptomic characterization, different molecular subtypes of GBM have been identified. The original classification in four subtypes proposed by Verhaak, neural (N), classical (CL), proneural (PN), and mesenchymal (MES) [148], was updated and recent studies reclassified GBMs in PN, CL, and MES subtypes [149,150]. Among them, PN has been associated with a better outcome, whereas MES to poor survival [151,152]. Intriguingly, survival analysis in the three GBM subtypes showed that high expression of TG2 significantly correlates with a poor clinical outcome in the cohort of MES GBMs (Figure 3).

MES GBM patients exhibit a high degree of necrotic areas and hypoxic regions. It has been reported that TG2 is highly expressed in the MES subtype, either in GBM patients and or in GBM stem-like cells (GSCs), and its expression is enriched in perinecrotic and hypoxic regions of GBM tissues [143,147]. In this context, high levels of TNF-α promote NF-κB-induced expression of TG2, which in turn activates master transcription factors of the MES subtype, such as c/EBPβ [143]. Furthermore, Ganesh and colleagues proposed an interactive/regulatory role for the G protein-coupled receptor 56 (GPR56/ADGRG1) on TG2 expression, both at the transcriptional and post-translational level, during the PN to MES transition process in GBM. The inverse correlation of GPR56 and TG2 expression, along with the extracellular signaling induced by hypoxia in the MES subtype, may contribute to the PN to MES transition through both the ECM remodeling mediated by the open transamidase-active conformation of TG2 and TG2-mediated NF-κB pathway activation [147]. The relationship between TG2 and GBM MES subtype has been further corroborated by two studies showing that TG2 correlates with the expression of MES GSC markers [24,146]. It has been reported that TG2 is highly expressed in CD44^high^ GBM and CD44^+^ MES GSCs, promoting GSC proliferation by inducing the expression of inhibitor of DNA-binding 1 protein (ID1) [24]. On the other hand, Sullivan and colleagues demonstrated that the MES GSC marker aldehyde dehydrogenase 1A3 (ALDH1A3), which catalyzes the conversion of retinhaldehyde to RA, induces the expression of TG2 through the production of RA [146]. Furthermore, TG2 is overexpressed in radioresistant MES GSCs and it has been observed that irradiation-induced TG2 expression. This finding supports the hypothesis that therapy-induced MES subtype transdifferentiation is the major cause of acquired resistance to conventional therapy [143].

The role played by TG2 in radio- and chemotherapy resistance has been described in several types of tumors including GBM [154]. However, the molecular mechanisms through which TG2 induces resistance are not well elucidated. Emerging studies demonstrated that TG2 could exert a protective effect against radiation directly through its expression in tumor cells or indirectly through its secretion in the tumor microenvironment (TME) [143,155,156,157]. Recently, it has been demonstrated that TG2 enhances radio resistance in GBM by promoting the maintenance of autophagic flux [157,158]. After irradiation, TG2 interacts with other proteins including syndecan 1 (SDC1), flotillin 1 (FLOT1), and betaine homocysteine methyltransferase (BHMT), forming protein complexes that promote the fusion of autophagosomes with lysosomes enhancing the level of autophagy [157]. In addition, TG2 together with SDC1 can also facilitate the fusion of autophagosomes with lysosomes by coordinating the binding of EPG5 to LC3 and subsequently stabilize the STX17-SNAP29-VAMP8 QabcR SNARE complex assembly [158]. TG2 is also involved in the crosstalk between non-coding RNAs (ncRNAs) that modulate the radio resistance of GBM cells. The lncRNA HOTAIRM1 contributes to radio resistance by the modulation of the reactive oxygen species (ROS) levels and mitochondrial function by regulating TG2 expression, potentially via acting as a sponge of miR-17-5p [155]. Moreover, as mentioned above, TG2 could also exert a radio-protective effect by an indirect mechanism, through its secretion by cells of TME. Intriguingly, it has been demonstrated that after irradiation, astrocytes secrete TG2 into the ECM, generating a tumor-supportive microenvironment that protects glioma cells from radiation [156].

TG2 expression has also been associated with chemotherapy resistance [14,159]. Dyer and colleagues tested whether TG2 expression was implicated in conferring resistance to several chemotherapeutic drugs such as lomustine, doxorubicin, TMZ, vincristine, cisplatin, and cyclophosphamide. However, it does not confer resistance to all chemotherapeutic drugs, commonly used for brain tumors. Particularly, in the U118MG GBM cell line, TG2 expression promotes resistance to lomustine but not to TMZ [23], whereas Yuan and colleagues demonstrated that inhibition of TG2 enhanced sensitivity to carmustine [160,161]. Treatment of GBM cell lines with the competitive TG2 inhibitor monodansylcadaverine (MDC) or the selective small molecule irreversible TG2 inhibitors, KCA075 or KCC009, in combination with carmustine, enhanced cell death. Treatments with TG2 inhibitors induced a shift toward a pro-apoptotic profile in GBM cell lines, with an enhancement in the levels of pro-apoptotic BH-3only protein, BIM, and a reduction of levels of anti-apoptotic proteins (i.e., survivin, phosphorylated Akt, Bad, and GSK-3β). These findings were also confirmed in the subcutaneous GBM xenograft mouse model [160]. Similarly, it has been demonstrated that KCC009 increased the sensitivity to carmustine in vitro and in vivo. KCC009 disrupted fibronectin assembly in the ECM, interfering with its pro-survival characteristics [161]. Recently, in contrast with the findings of Dyer and colleagues, it has been reported that biomimetic nanocomplexes containing TG2 small interfering RNA (siRNA) can sensitize U87MG cells to TMZ increasing apoptosis [162].

## 6. TG2 in Other CNS Tumors

To date, the role of TG2 has been investigated only recently in MB, an embryonal tumor of the CNS. Particularly, Marquardt and colleagues proposed the contribution of TG2 in tumor inflammation as a potential resistant mechanism in MYC-driven MB [163]. They showed the upregulation of TG2 after class I HDAC inhibitor treatment of MYC-driven MB cell lines, as direct effect of NF-κB activation, leading to a pro-inflammatory cytokine response and macrophage infiltration [163].

High TG2 activity and expression were reported in a series of meningiomas including WHO grades 1–3, suggesting a potential role of TG2 in meningioma tumorigenesis [22]. Similarly to previous results, Huang and colleagues reported that TG2 was highly expressed in a collection of twenty-four cell cultures derived from meningioma patients, and these data were subsequently corroborated in an independent set of eighty-two meningiomas by immunohistochemical analysis. Intriguingly, high TG2 expression was correlated with higher WHO grading as well as meningioma relapse [164]. Harb and colleagues also confirmed this finding [165]. In addition, it has been reported that high TG2 expression was positively associated to high Ki67 labeling index, the presence of residuals after surgery, high incidence of relapse after surgery, meningioma progression to higher grades and with relapse-free survival (RFS) [165]. All these findings proved that TG2 could represent a potential candidate to predict risk of meningioma relapse and progression into higher WHO grades. Moreover, the role of TG2 in promoting tumor growth and in protecting from radiation therapy has also been demonstrated [22,164]. As reported in GBM, TG2 interacts with fibronectin remodeling ECM and mediating critical pro-survival interaction [22,161]. Similarly, immunohistochemical analysis showed the colocalization of TG2 and fibronectin in meningioma tumor samples [22]. Inhibition of TG2 using KCC009, induced a reduction in the disposition and assembly of fibronectin in the ECM. Moreover, as reported in GBM, KCC009 treatment increased the sensitivity of meningioma cell lines to radiation [22,161]. These preliminary results suggest that TG2 could be an interesting target for developing more efficient treatment options for patients with high-grade or relapsed meningioma.

## 7. TG2 in Neuroblastoma

The role of TG2 in NBL, which represents the most common extracranial solid tumor of the sympathetic nervous system and accounts for about 8% of malignancies of the pediatric age [166], has been deeply investigated. Moreover, as previously stated, NBL cell lines (i.e., SH-SY5Y cells) under differentiating stimuli are usually considered a neuronal model [86]. Melino and colleagues provided one of the first evidence demonstrating a possible involvement of TG in NBL differentiation [167]. Indeed, they established that the differentiating effect of RA was associated with an increase in TG activity and a parallel transient increase in polyamine levels (PUT and SPD). The same research group also showed that the expression of TG2 in the SK-N-BE(2) neuroblastoma cells was correlated with the rate of apoptosis and that the protein was specifically expressed in the apoptotic bodies [168]. It is interesting to note that, as previously stated, SK-N-BE(2) cell line, similarly to other NBL cells established in vitro, is characterized by the presence of three distinct phenotypes called S-type (flat substrate-adherent and non-invasive cells with glial precursor features), N-type (cells with neuronal characteristics), and I-type (cells with intermediate morphology) [117,118]. According to Piacentini and coworkers [169], RA exposure increased TG2 expression and apoptotic index in the S-type SK-N-BE(2) clone, but not in the N-type variant. Moreover, the overexpression of TG2 in the same cellular model caused a rapid increase in apoptotic rate. On the contrary, the reduction of TG2 expression by plasmids containing cDNA in antisense orientation resulted in a significant decrease in cell death [170]. The role of TG2 in induction of apoptosis, and its S- and N-type specific expression, was also confirmed in in vivo model [171]. The TG2-dependent increase in apoptosis in SK-N-BE(2) cells is due to the TG2-mediated modification of proteins involved in mitochondrial homeostasis, including pro-apoptotic members of the Bcl-2 family [172,173]. Furthermore, starting from the observation that RA-dependent induction of TG2 did not produce an increase in apoptotic rate in human neuroblastoma SH-SY5Y cells [174], Tucholski and Johnson demonstrated that the pro-apoptotic role of TG2 was dependent on the type of stressors and on how the transamidation activity of the enzyme is affected [175]. In fact, apoptotic stimuli (e.g., osmotic stress) that increase Ca2+ intracellular levels are linked to pro-apoptotic effects in SH-SY5Y cells [175]. On the contrary, TG2 shows anti-apoptotic properties in the absence of transamidation activity (heat shock- or DNA-damage-induced stress) [171,176]. These studies have contributed to a better understanding of the role of TG2 in the induction of apoptosis, depending on the cellular model, the type of stimuli, but also on the intracellular localization of the enzyme [39,74].

In particular, although TG2 is mainly present in the cytoplasm, it can also be found in the nucleus, mitochondria, in the extracellular compartment (in the plasma membrane or in ECM) [74,177]. At extracellular level, it has been known that TG2 interacts with ECM proteins through its enzymatic (crosslinking activity) and non-enzymatic mechanisms (forming a heterocomplex between fibronectin and integrin) [75]. Moreover, extracellular TG2 interacts with several growth factor receptors (tyrosine kinase proteins), including platelet-derived growth factor (PDGF), vascular endothelial growth factor (VEGF), and epidermal growth factor (EGF) receptors, activating cell survival signaling pathways [177]. In nervous system, the nerve growth factor (NGF) and its tyrosine kinase receptor TrkA play a key role in regulating neuronal function [178], and NGF has also been shown to induce cell differentiation in Neuro2a NBL cells through increased expression and transamidation activity of TG2 [179]. This result was confirmed by the study of Algarni and coworkers, which also reported a TG2-mediated amine incorporation and protein crosslinking activity in Neuro2a and human SH-SY5Y cells after NGF treatment in a time- and dose-dependent manner [180].

As previously stated, in several NBL cell lines RA strongly induces differentiation toward a neuronal-like-phenotype [181], so much that the use of RA analogs has been proposed in NBL therapy [182]. It is therefore clear that, since the transamidation activity of TG2 plays a key role in the response of NBL to retinoids, the switch from the Ca^2+^-bound to the GTP-bound conformations of TG2 (also depending on cell-specific TG2 isoforms) represents a crucial step. In particular, *TGM2* alternative splicing emerges as one of the mechanisms that increase cell-type-dependent TG2 functional complexity, and whose dysregulation seems to play a role in several pathologies including cancer and neurodegeneration [60,64,183,184]. Tee and colleagues clearly demonstrated that in NBL cells all-*trans* RA induce the expression of both TG2-L and TG2-S isoforms, which however showed opposing effects in cell differentiation [185]. Indeed, since TG2-S lacks the R580 (a key residue in the C-terminus for the GTP-binding regulatory domain), its constitutive activation of transamidase catalytic property contributes to NBL cell differentiation. On the contrary, TG2-L represses NBL differentiation and consequently promotes cell growth [185]. Similarly, it has been demonstrated that different effects of TG2-S and TG2-L isoforms in response to hypoxia have been associated with *MYCN* amplification [186]. Moreover, it has been suggested that the different response to all-*trans* RA in NBL cells may depend on different regulation of *TGM2* transcripts by TG2-lncRNA and LEV [70].

## 8. Discussion and Conclusions

TG2 is a ubiquitous enzyme whose activity has been reported in the various cell types of the CNS [4,7]. Due to its involvement in the physiological regulation of several processes in the neural tissues, it is not surprising that TG2 dysregulation contributes to the pathogenesis of neurodegenerative and neuroinflammatory diseases, as well as to the progression of nervous system tumors [19,104,106]. Different studies investigated the potential use of TG2 inducers and/or inhibitors as new therapeutic tools to treat such diseases [11,55,92,156]. As described above, different molecular factors are able to induce *TGM2* expression, such as RA and mediators of inflammation, and the mechanisms underlying their action have been widely investigated [51]. On the other hand, several examples of TG2 inhibitors have been reported, such as competitive amines and reversible and irreversible inhibitors, but their effects on TG2 pleiotropic functions are more complex to outline [11,92,94]. Representative inhibitors of TG2 are summarized in Table 1.

Although the application of TG2 inhibitors to mouse models of Huntington’s and Parkinson’s diseases showed promising results, this was not the case when translated into clinical trial studies [11]. Encouraging results emerged from considering TG2 inhibition for the treatment of multiple sclerosis, particularly interfering with fibronectin assembly in the ECM by using KCC009 [199,200]. Interestingly, it has been reported that the same selective small molecule irreversible TG2 inhibitor, in combination with carmustine, enhanced cell death in GBM cells and xenograft mouse models [160] and increased the sensitivity of meningioma cell lines to radiation [22,161]. Likewise, the strategy for sensitizing GBM to radiotherapy using the TG2-specific inhibitor GK921 showed promising results [143]. The same strategy was reported by Sullivan and colleagues, showing the benefits of combining the TG2 inhibitors Z-Don and MDC with the current standard of care for GBM [146]. Intriguingly, treatment with these inhibitors affects the self-renewal, proliferation, and survival of the aggressive MES GSCs [146]. Likewise, Fu and colleagues reported the effects of MDC on CD44^high^ GSCs, both in vitro and in vivo [24]. In addition, the small molecule TG2 inhibitor TTGM5826, through stabilizing the cytotoxic open state conformation of TG2, impaired the growth of different cancer cells, including GSCs [192]. Therefore, further studies on the mechanisms and the effects of TG2 inhibitors in nervous system tumors could be necessary in order to exploit their potential therapeutic application.

From the same perspective, the interaction between TG2 and non-coding RNAs in nervous system tumors could be interesting to investigate. Indeed, its potential impact on TG2-related tumorigenic processes emerged in terms of both direct and indirect regulation of *TGM2* expression, affecting TG2 functions and tumor-associated processes [69,70,71,155]. On the other hand, therapeutic targeting of the cellular components that promote the disease within its microenvironment may be favorable to enhance the efficacy of standard-of-care treatment. The understanding of the mechanisms underlying the effects of TG2 secretion by endothelial cells, astrocytes, and microglia, among others, could allow the identification of novel therapeutic strategies [55,104,156]. In this context, it could be interesting to explore in depth the role of TG2 in angiogenesis of nervous system tumors. It has been reported that extracellular TG2 is involved in matrix-bound VEGF-mediated angiogenesis, suggesting that inhibition of TG2 could be a potential therapeutic target in the treatment of angiogenic pathologies [201]. Therefore, since GBM is a highly vascularized tumor characterized by a high expression of VEGF [202], targeting extracellular TG2 activity might be hypothesized as alternative strategy to overcome the mechanisms adopted by GBM to develop resistance to anti-angiogenic therapy [203].

Given the involvement of TG2 on several processes, i.e., radio- and chemoresistance, inflammation and differentiation, thus contributing to nervous system tumor pathogenesis, it becomes more and more interesting to develop efficient approaches to target this enzyme.

## Figures and Tables

**Table 1:** Representative TG2 inhibitors.

Inhibitor	Type	References
MDC	Pseudo-substrate; competitive	[187]
CP4d	Reversible	[188]
GK921	Reversible	[143,189,190]
LDN-27219	Reversible	[191]
TTGM5826	Reversible	[192]
Cystamine	Pseudo-substrate; irreversible	[193,194]
KCC009	Peptidomimetic; irreversible	[14,22,160,161]
Z-DON (Z006)	Peptidomimetic; irreversible	[14,180,192,195,196]
ZED1227	Peptidomimetic; irreversible	[197]
NTU283 (D003)	Irreversible	[198]

## Data Availability

Data are contained within the article.
